# Safety and Efficacy of Microinvasive Glaucoma Surgery

**DOI:** 10.1155/2017/3182935

**Published:** 2017-04-23

**Authors:** David Z. Chen, Chelvin C. A. Sng

**Affiliations:** ^1^Ophthalmology Department, National University Hospital, Singapore; ^2^Glaucoma Service, Moorfields Eye Hospital, London, UK; ^3^Singapore Eye Research Institute, Singapore

## Abstract

Microinvasive glaucoma surgery (MIGS) is emerging as a new therapeutic option for glaucoma patients who wish to reduce their medication burden and avoid the postoperative complications of conventional glaucoma filtration surgery. These devices differ in terms of their efficacy and safety profile. Schlemm's canal devices have the most favorable safety profile at the compromise of modest efficacy, while subconjunctival and suprachoroidal devices are potentially more effective at lowering the intraocular pressure at the expense of a higher rate of complications. This review consolidates the latest evidence on the efficacy and safety of the MIGS devices in clinical use and provides an overview on upcoming devices which would likely also become viable treatment options in the near future. These clinical data would assist a glaucoma surgeon in selecting the most appropriate MIGS device for each patient based on the glaucoma severity and patient expectations.

## 1. Introduction

The management of glaucoma revolves around control over intraocular pressure (IOP). Traditionally, this has been achieved through both nonsurgical (topical medications [1] or laser therapy [2, 3]) and surgical (trabeculectomy, glaucoma drainage devices) means [4–6]. Neither methods are ideal—compliance with medications being an issue for the former while surgical complications are common in the latter.

More recently, microinvasive glaucoma surgery has emerged as a new treatment for open-angle glaucoma (OAG) including pseudoexfoliative glaucoma (PXF) and pigmentary glaucoma (PG). MIGS typically utilizes an ab interno approach, often through a clear corneal incision familiar to most ophthalmic surgeons, thus allowing for minimal tissue disruption, a more favorable risk profile, and faster recovery as compared to conventional trabeculectomy or glaucoma drainage device implantation. Currently, it is most commonly indicated for mild-to-moderate glaucoma in patients with poor tolerance or compliance to antiglaucomatous medications. However, critics question the poorer IOP-lowering effect of MIGS. At the point of writing, there are a multitude of MIGS devices, with several different mechanisms of action, and varying efficacy and safety profiles. In this review article, we attempt to review current understanding on MIGS and consolidate the safety and efficacy of these devices.

## 2. Material and Methods

A PubMed search using a combination of keywords and Medical Subject Headings on the following keywords was made on 29th December 2016: “safety”, “complication”, “efficacy”, “outcome”, “minimally invasive glaucoma surgery”, “micro-invasive glaucoma surgery”, “MIGS”, “trabectome”, “iStent”, “iStent inject”, “Hydrus”, “excimer laser trabeculotomy”, “ELT”, “CyPass”, “XEN”, “Aquesys”, “Innfocus”. Results were classified according to the types of device (Schlemm's canal, suprachoroidal, and subconjunctival devices). All randomized controlled trials (RCTs) and case series in English-published articles either online or in print prior to the date of search were considered. Prospective articles were given higher priority. Review articles were excluded from the search, but relevant referenced primary articles were included in this literature review. For articles where full texts were unavailable, we requested for a full article from the corresponding author; failing which, relevant data was extracted from the abstract itself.

The primary outcome measures for efficacy included preoperative and postoperative IOP, as well as the number of medications required. Success and failure rates were defined heterogeneously across studies, and only selected results from RCTs were included as a secondary outcome measure for efficacy. Primary outcome measures for safety included a qualitative description of intraoperative and postoperative complications, as well as the need for repeat surgeries.

## 3. Results

**Figure 1:**
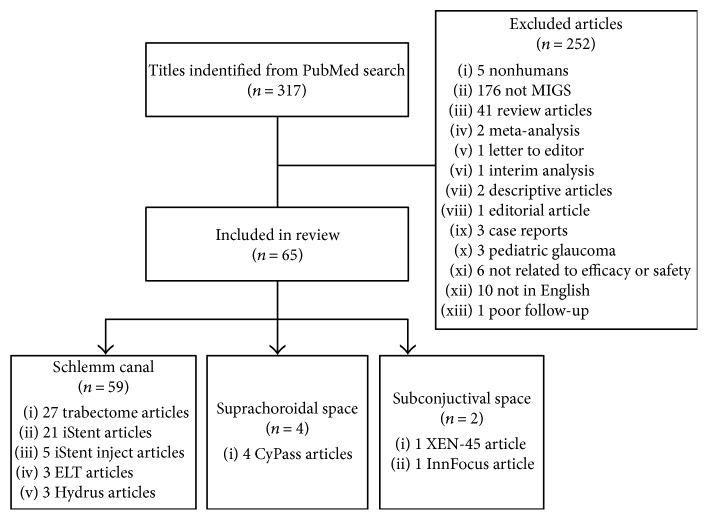
Literature search and included articles.

A total of 317 articles were included from the search result. After review and exclusion by a single reviewer, 65 articles were included (Figure 1). The following sections summarize the findings.  Table 1 summarizes the physical properties of these devices and their approved usage in the US as well as in Europe.

### 3.1. Schlemm's Canal

Schlemm's canal devices are performed through an ab interno method through the assistance of a gonioscopy lens, with an aim to increase aqueous outflow through the conventional pathway. Therefore, the potential effect on aqueous outflow is influenced by episcleral venous pressure. The commonest procedures involving the Schlemm's canal include the removal of trabecular tissue (ab interno trabeculotomy, excimer laser trabeculotomy) or the implantation of a small device (e.g., iStent, iStent inject®, and Hydrus MicroShunt). The following sections would highlight the different methods in detail.

#### 3.1.1. iStent

The iStent (Glaukos Corporation, CA, USA) is a heparin-coated titanium microbypass device. This L-shaped device is 1.0 mm long and 0.33 mm wide and comes preloaded in a single-use sterile inserter. The short side (inlet) faces the anterior chamber, while the lumen (foot) resides in the Schlemm's canal, and could be inserted either right or left handedly. In 2012, this device was approved in the US for use in patients with mild-to-moderate glaucoma who are undergoing cataract surgery. It was the first MIGS device to be approved for use in the US. In Europe, this device is approved for use either as a stand-alone surgery or as a surgery combined with cataract surgery.

Several authors have independently reviewed the efficacy of iStent devices (Table 2). When performed as a stand-alone procedure, the implantation of a single iStent reduced IOP by 4.2 mmHg after 18 months as compared with medicated baseline IOP in a RCT performed by Katz et al. [7]. In a retrospective analysis of 42 pseudophakic eyes by Ferguson et al., the implantation of a single iStent reduced IOP from 20.26 ± 6.00 mmHg preoperatively to 13.62 ± 4.55 mmHg at two years postoperatively (*p* < 0.01) [8]. However, there was no significant decrease in the number of medications (1.95 ± 1.01 versus 1.33, *p* > 0.05), and there was a high dropout rate of 50% (21 of 42 patients). Buchacra et al. demonstrated an absolute IOP reduction of 9.5 mmHg from baseline (relative reduction of 36%) in a small group of 10 subjects with secondary OAG [9], but this finding might be limited by the small sample size and relatively short follow-up period of one year.

The placement of more iStents in the same eye appears to have an additive effect on IOP lowering; Katz et al. demonstrated a progressively higher absolute IOP reduction from 4.2 mmHg with one iStent to 8.3 mmHg with three iStents at 18 months postoperatively. Other studies which placed two iStents in the same eye reported an absolute IOP reduction of between 5 and 8 mmHg [7, 10–16]. Vold et al. reported an IOP reduction of 11 mmHg when subjects with newly diagnosed open-angle glaucoma were implanted with two iStents [10, 11, 15].

iStent is also commonly performed with cataract surgery. A large RCT performed by Craven et al. demonstrated modest IOP reduction of 1.5 mmHg from preoperative medicated IOP levels with a corresponding decrease in medications from 1.6 ± 0.8 to 0.3 ± 0.6 [17], while another large RCT by Samuelson found an equally modest IOP reduction of 1.5 mmHg after 1 year with a decrease in medication from 1.5 ± 0.6 to 0.2 ± 0.6 [18]. Two other smaller RCTs by Fea et al. found similar IOP reductions and medication reduction to 0.5 medication or less [19, 20] (Table 2). In some RCTs, there was a specified medication washout period postoperatively and the authors found postoperative IOP to be around 17 mmHg [10, 16]. In contrast, other prospective and retrospective case series have found inconsistent IOP reductions from the medicated baseline, ranging from as low as 1.6 mmHg at 6 months to as high as 9.2 mmHg at 3 years postoperatively [14, 21–27]. This suggests that while combined cataract surgery and iStent may have synergistic effects in lowering IOP, their effects are not necessarily additive.

Intraoperative and postoperative complications are listed in Table 3. In general, iStent implantation is associated with a good safety profile that is comparable with cataract surgery alone, with the commonest complication being transient (and commonly self-limiting) hyphema. Stent obstruction and stent malposition also occur and often do not require intervention, though sometimes they may require laser or surgical intervention [17, 18, 27] (Table 3). Reoperation rates were understandably rare as the subjects who underwent iStent implantation usually had mild or moderate glaucoma.

#### 3.1.2. iStent Inject

The iStent inject, the second generation version of the iStent, is a single-piece heparin-coated, gamma-sterilized titanium device. It is symmetrically designed, and up to two iStents can be injected with a single injector device using the G2-M-IS injector system. This allows for the availability for the surgeon to inject two iStents while entering the eye only once, thus reducing the risks of adverse events even further.

Fea et al. compared the efficacy of two iStent inject implantations with fixed combination of latanoprost/timolol medication administration in a randomized unmasked study [28]. At one year postoperatively, the medicated IOP of the iStent inject group has decreased from 21.1 ± 1.7 mmHg (postwashout baseline IOP 25.2 ± 1.4 mmHg) to 13.0 ± 2.3 mmHg and concluded that the effect of two iStent injects is at least as effective as two medications while reducing medication burden. Two other independent prospective studies by Voskanyan et al. [29] and Arriola-Villalobos et al. [30] found similar results (Table 2).

Gonnermann et al. evaluated the only comparative study available between Trabectome and iStent inject [31]. In a retrospective intraindividual comparative study, the authors performed Trabectome and cataract surgeries in one eye and two iStent inject implantations with cataract surgery in the contralateral eye. At one year postoperatively, the authors found similar IOP-lowering effects in both groups and concluded that both were effective in lowering IOP (Table 2). The safety profiles of both groups were also similar.

#### 3.1.3. Hydrus Microstent

The Hydrus® (Irvine, CA, USA) is a relatively new Schlemm's canal device. Using a customized preloaded hand-held injector, the 8 mm long crescent-shaped device is implanted ab interno; its curvature shaped to match that of the Schlemm's canal. It dilates the canal up to 3 clock hours and allows direct communication between the anterior chamber and the Schlemm's canal.

A recent randomized controlled trial, HYDRUS II [32], recruited 50 subjects to compare the safety and efficacy of the Hydrus microstent (Hydrus) in a combined Hydrus and phacoemulsification procedure with a control population of 50 subjects with OAG who underwent only phacoemulsification, and they were followed up for two years postoperatively. For the combined group, postwashout IOP levels decreased from 26.3 ± 4.4 mmHg preoperatively (medicated IOP 18.9 ± 3.3 mmHg) to 16.9 ± 3.3 mmHg postoperatively (Table 2), and the number of medications required also reduced from 2 ± 1 to 0.5 ± 1. While the mean postoperative IOP was lower than baseline IOP for both groups at 12 and 25 months postoperatively, the postoperative IOP of the combined group was significantly lower than that of the control group at 24 months (16.9 ± 3.3 mmHg versus 19.2 ± 4.7 mmHg, *p* = 0.0093). Postoperatively, the proportion of subjects with a 20% or greater reduction in washed out diurnal IOP compared with baseline was significantly higher in the study group than in the control group (80% versus 46%, *p* = 0.0008).

Fea et al. conducted a nonrandomized prospective interventional case series between 31 subjects who underwent Hydrus implantation and 25 subjects who underwent selective laser trabeculoplasty (SLT) to compare the IOP-lowering effects between the two groups [33]. At one year postoperatively, mean IOP decreased from 23.1 ± 5.08 to 16.5 ± 2.6 mmHg for the Hydrus group, and a similar decrease was found for the SLT group (from 23.2 ± 2.15 mmHg to 15.9 ± 2.49 mmHg). However, subjects in the Hydrus group had significantly greater medication reduction when compared to those in the SLT group (−1.4 ± 0.97 versus −0.5 ± 1.05, *p* = 0.001).

The Hydrus implant is generally safe, and complications are infrequent. Several studies have shown the Hydrus to have a transient early IOP spike from baseline in less than 10% of patients [32–34], of which one of the authors attributed to retain viscoelastic material at the end of operation [33]. Gandolfi reported transient hyphema in 4 subjects (19.0%) in a retrospective cohort of subjects, and this resolved without treatment [34]. The HYDRUS II study found focal peripheral anterior synechiae in 9 subjects (18.8%), which did not require further intervention [32].

#### 3.1.4. Ab Interno Trabeculotomy

Ab interno trabeculotomy is performed most commonly using the Trabectome device (NeoMedix, Tustin, USA). Using high frequency electrocautery energy, the Trabectome ablates part of the nasal trabecular meshwork and inner walls of the Schlemm's canal over a 90° to 120° arc through a single temporal incision [35]. This procedure may be performed with or without phacoemulsification in the same setting.

Numerous studies have been conducted to identify the IOP-lowering effect of Trabectome, either as a stand-alone procedure or as a procedure combined with phacoemulsification. However, results from prospective RCTs are lacking. The first prospective case series was performed by Minckler et al. in 2005 [35], who recruited 37 subjects for stand-alone Trabectome procedure and followed them up for one year postoperatively. They found postoperative-medicated IOP to be 16.3 ± 2.0 mmHg, down from a baseline postwashout IOP of 28.2 ± 4.4 mmHg. There was a corresponding decrease in medication usage from 1.2 ± 0.6 at baseline to an average of 0.3 medications through the postoperative period.

Subsequent studies have found similar IOP-lowering effects between 4 mmHg and 10 mmHg up to one year postoperatively [36–40] (Table 2). The effect appears to be more significant in patients with exfoliative glaucoma. In a large prospective cohort study done by Ting et al. [36], subjects with exfoliative glaucoma experienced a greater IOP reduction postoperatively compared to subjects with open-angle glaucoma one year after Trabectome incision with or without phacoemulsification (Table 2). The authors postulated that the mechanical effect of trabecular meshwork removal facilitates the washout of exfoliative material thus contributing to a great decrease in IOP.

Being a minimally invasive procedure, the safety profile of Trabectome is impressive (Table 3). A prominent observation of using the Trabectome is blood reflux into the anterior chamber. Almost all patients experience some degree of blood reflux upon the withdrawal of the instrument from the eye, although the effects are almost always self-limiting [35, 40–43]. Intraoperative blood reflux is often considered to be a positive sign which indicates patency of the Schlemm's canal and the downstream collector channels and aqueous veins [42], although inadvertent damage to anterior chamber structures may also occur, resulting in the formation of peripheral anterior synechiae or goniosynechiae [35]. Overall, the complication rates of Trabectome surgery are much lower than those of traditional glaucoma filtration surgeries.

#### 3.1.5. Gonioscopy-Assisted Transluminal Trabeculectomy, Kahook Dual Blade, and Trab360

The following devices are similar to the Trabectome and increase outflow through the Schlemm's canal through directly removing a part of the trabecular meshwork. As such, some may argue that they may not qualify as MIGS because there is significant tissue destruction. The Kahook Dual Blade (DKB) (New World Medical, CA, USA) is a novel dual-blade device that elevates and removes the trabecular meshwork, allowing for cleaner removal of the tissue, thus minimizing damage to adjacent structures [44]. There is limited clinical data on the efficacy of this FDA-approved device. Gonioscopy-assisted transluminal trabeculotomy (GATT) (Glaucoma Associates of Texas, TX, USA) and Trab360 are devices that allow circumferential 360-degree removal of the trabecular meshwork. Studies suggest that the efficacy of these procedures is superior to that of Trabectome, but with a higher rate of hyphema [45–47].

#### 3.1.6. Excimer Laser Trabeculotomy

Unlike the Trabectome, excimer laser trabeculotomy (ELT) utilizes an endoscopically guided excimer laser (AIDA, TuiLaser, Munich, Germany) to induce microperforations within the trabecular meshwork [48]. The photoablative effects of the XeCl 308 nm laser open the trabecular meshwork without thermal effects which may induce scarring. This procedure may be performed with or without phacoemulsification.

In 2006, Wilmsmeyer et al. published a retrospective review on a group of 135 patients with open-angle glaucoma [48]. 75 patients underwent ELT alone (group 1), while another 60 patients with concurrent visually significant cataracts underwent combined phacoemulsification and ELT (group 2). At one year postoperatively, there was a reduction of IOP from 23.3 ± 0.6 mmHg to 18.8 ± 0.8 mmHg for group 1 and a corresponding reduction of IOP from 22.4 ± 0.8 mmHg to 16.4 ± 0.4 mmHg for group 2 (Table 2). The number of medications required was not significantly different pre- and postoperatively for both groups. The authors concluded that phacoemulsification with ELT is more efficacious than ELT alone. Babighian et al. conducted a prospective RCT comparing ELT with selective laser trabeculoplasty (SLT) in 15 patients with refractory open-angle glaucoma for two years postoperatively [49]. In the ELT group, there was a significant decrease in IOP from 25.0 ± 1.9 mmHg preoperatively to 17.6 ± 2.2 mmHg at two years postoperatively, with a corresponding decrease in medications from 2.27 ± 0.6 to 0.73 ± 0.8 (Table 2). These results were similar to those of SLT, and the authors also found no significant difference in the success rates between the two groups. Another similar study by Bagighian et al. in 2006 found similar findings [50].

Complications from ELT are mostly self-limited. In Babighian's study, 80% of patients had transient intraoperative anterior chamber bleeding and 20% of patients had IOP increase of more than 5 mmHg which resolved spontaneously without treatment (Table 3). No reoperations were reported. In contrast, Wilsmeyer et al. reported reoperation in 19% of their patients, most of them due to treatment failure (Table 3). Two patients had iris adhesion to corneal tunnel, and one patient had central retinal vein occlusion five months postoperatively, which the authors deemed to be unrelated to the procedure [48].

### 3.2. Suprachoroidal Space

In contrast to Schlemm's canal devices, several other devices have attempted to utilize the alternative uveoscleral outflow pathway as a means to reduce intraocular pressure. Unlike the Schlemm's canal in which aqueous outflow could be affected by episcleral venous pressure, the suprachoroidal space is a potential space that confers minimal resistance to aqueous outflow. It allows aqueous to traverse the sclera directly via the intercellular spaces between ciliary muscle fibres and loose connective tissues of the suprachoroidal space. The CyPass MicroStent (Transcend Medical, Menlo Park, CA, USA) and the iStent Supra (Glaukos Corporation, CA, USA) are MIGS devices that drain to the suprachoroidal space. Other devices that utilize this drainage pathway include the Gold Micro Shunt (SOLX Inc., Waltham, MA, USA) and the Aquashunt, but these require ab externo implantation requiring conjunctival dissection and scleral incision, hence are not typically regarded as MIGS devices and are beyond the scope of this review.

#### 3.2.1. CyPass

The CyPass MicroStent (CyPass) is a flexible fenestrated microstent made of polyimide material, which follows the curvature of the sclera as it is threaded through a guidewire and applicator into the supraciliary space. This is performed through a single corneal incision and blunt dissection through gonioscopic guidance.

Vold et al. published the only RCT to date for the CyPass, the COMPASS II study [51]. This large RCT included 374 subjects with OAG who underwent combined phacoemulsification and CyPass implantation, compared with 131 control subjects who underwent standard phacoemulsification alone. Subjects were followed up for two years postoperatively with less than 5% dropout rate. IOP reduced from preoperative washout levels of 24.4 ± 2.8 mmHg to 17 ± 3.4 mmHg at two years postoperatively. Medication requirement was reduced from an average of 1.4 ± 0.9 to 0.2 ± 0.6 mmHg (Table 2). Compared with the control group which had only phacoemulsification, there was a significantly greater IOP-lowering effect as well as threefold reduction of IOP-lowering medications in the group which underwent combined phacoemulsification and CyPass implantation. The authors concluded that CyPass with phacoemulsification had sustained 2-year efficacy benefit for the IOP control. This comprehensive study corroborated the efficacy findings in previous prospective case series and interventional studies by other authors [52–54] (Table 2).

Compared to Schlemm's canal devices, the CyPass is associated with a higher incidence of early IOP fluctuations (both transient hypotony and transient ocular hypertension) in the immediate postoperative period [51–54]. Transient hypotony is hypothesized to be due to the creation of a cyclodialysis cleft which might extend beyond the external diameter of the CyPass [52]. Unlike in subconjunctival procedures, transient hypotony does not cause anterior chamber shallowing as aqueous outflow is contained internally and ocular tissue integrity is maintained. On the other hand, transient IOP spikes could potentially be dangerous for patients with advanced glaucoma; unlike in subconjunctival drainage devices where outflow could be modulated with mitomycin C (MMC) or bleb manipulation procedures such as needling, there is no way of preventing or reversing scarring in the suprachoroidal space. Overall, however, reported adverse event rates of CyPass with phacoemulsification were not significantly higher than those of phacoemulsification alone (39% versus 36%, resp., COMPASS II study) [51]. Being an ab interno procedure, the CyPass also eliminates concerns about bleb-related and conjunctival complications.

#### 3.2.2. iStent Supra

The iStent Supra is an investigational device similar to the CyPass. It is made of polyethersulfone and titanium and implanted ab interno; it may be implanted after cataract surgery. Studies are underway to evaluate the efficacy and safety of this device, though there is no published literature at the point of writing.

### 3.3. Subconjunctival Space

The subconjunctival space is a potential space under the Tenon's capsule which is not part of the physiological outflow pathway. However, it is the drainage pathway most familiar to glaucoma surgeons as it is utilized in conventional glaucoma surgery, including trabeculectomy and tube implant surgery. Just like the suprachoroidal space, this area is not limited by the episcleral venous pressure but aqueous drainage can be compromised by fibrosis and scarring. The XEN-45® implant (Allergan, Dublin, Ireland) is the first MIGS device that drains to the subconjunctival space. Though the Innfocus MicroShunt (Santen Pharmaceutical Co. Ltd., Osaka, Japan) is implanted through an ab externo approach requiring conjunctival dissection, the US Food and Drug Administration (FDA) has classified it as a MIGS device; hence, it is included in this review.

#### 3.3.1. XEN-45 Gel Stent

The XEN-45 Gel Stent (XEN-45) is a hydrophilic collagen tube made with gelatin and glutaraldehyde. Its physical composition makes the device harder when dry, but softer and more flexible when hydrated. It is preloaded in an injector which allows controlled ab interno insertion into the subconjunctival space, emerging 3 mm posterior to the limbus. Bleb formation is confirmed at the end of surgery, and XEN-45 implantation may be performed together with cataract extraction surgery. The implant was recently approved by FDA for use in medically and surgically refractory open-angle glaucoma.

Recently, Perez-Torregrosa et al. published a prospective nonrandomized case series of 30 phakic subjects with OAG who underwent combined phacoemulsification and XEN-45 implantation [55]. One year after the surgery, IOP was reduced from 21.2 ± 3.4 mmHg preoperatively to 15.0 ± 2.47 mmHg postoperatively, with a corresponding reduction in medications from 3.07 ± 0.69 to 0.17 ± 0.65 (Table 2). The success rate was 90% (27 out of 30 eyes), with success being defined as IOP ≤ 18 mmHg without mediations.

The XEN-45 also demonstrated good safety profile in the same study above, with intraoperative hemorrhage (both intracameral and at scleral exit point) being the most common (Table 2). Importantly, postoperative encapsulation of filtration bleb was found in only one eye (3.3%), although a longer follow-up period may be required to identify the prevalence of late fibrosis of subconjunctival space. The authors also recommended that the optimal placement of the 6 mm device would be 2 mm subconjunctival, 3 mm intrascleral, and 1 mm intracameral to balance implant coverage and aqueous outflow. With an internal lumen diameter of 45 *μ*m and a length of 6 mm, the XEN-45 implant confers protection against hypotony as it has an intrinsic outflow resistance of 6–8 mmHg according to the Hagen-Poiseuille equation [56]. This is achieved through designing specific lengths and internal diameters of the tube, which has been demonstrated through flow testing by Sheybani et al. [57].

Aside from isolated case reports [58, 59] and a single pilot study on combined XEN implant with the Baerveldt tube (Abbott Inc., Lake Bluff, IL, USA) [60], there is currently a lack of other published literature on the XEN-45. A study by Sheybani et al. reported promising clinical results in 37 patients who underwent combined XEN-140 (140 *μ*m internal diameter) and XEN-63 (63 *μ*m internal diameter); implantation and cataract surgery, albeit with 9% of patients developing transient hypotony, require intracameral viscoelastic injections within the first postoperative week [61]. More randomized and controlled studies are required to determine whether the XEN-45 implant is as effective as its predecessors (XEN-140 and XEN-63) with a lower rate of hypotony.

#### 3.3.2. InnFocus MicroShunt

The InnFocus MicroShunt (MicroShunt) is an experimental device which is a trial product awaiting FDA approval. It is purported to act as a flow resistor to maintain the long-term transscleral pressure above 5 mmHg. As with all subconjunctival procedures, there exists a risk of subsequent conjunctival fibrosis limiting aqueous outflow [62]. For this reason, MicroShunt surgery may be performed with intraoperative MMC, as with XEN-45.

Batlle et al. studied the efficacy of the MicroShunt with MMC over a period of three years in a nonrandomized prospective case series of 23 eyes with OAG [63]. 14 subjects underwent isolated InnFocus insertion while another 9 had it implanted with concurrent cataract surgery. At the end of 36 months, there was a significant reduction in IOP from a preoperative level of 23.8 ± 5.3 mmHg to 10.7 ± 3.5 mmHg. The authors quote a qualified success rate of up to 95% up to three years (IOP ≤ 14 mmHg and IOP reduction ≥ 20%). In the same study, the authors found the complications to be transient and self-limiting (Table 3). Specifically, there were no cases of bleb leaks, infections, migrations, erosions, or other serious bleb-related complications known to conventional trabeculectomy.

However, the small and nonrandomized sample size of this study make their findings hard to generalize and more clinical studies would be required to support these preliminary findings. Our literature search did not reveal any other published articles on InnFocus MicroShunt; a multicenter clinical trial comparing MicroShunt to primary trabeculectomy is currently underway.

## 4. Conclusion

Currently, the glaucoma surgeon is spoilt for choice where MIGS devices are concerned. However, aside from the Trabectome and iStent, high quality evidence on the efficacy of MIGS devices is still lacking. The overall modest IOP reduction effect and generally favorable safety profile of Schlemm's canal devices make it a welcome option for patients with mild or moderate glaucoma who would like to reduce their medication burden. Suprachoroidal and subconjunctival devices offer the potential of greater IOP reduction, but suprachoroidal devices such as CyPass are potentially associated with unpredictable IOP spikes and hypotony while subconjunctival devices may fail as a consequence of subconjunctival fibrosis or result in bleb-related complications. More prospective randomized trials with longer follow-up periods are required to further evaluate the efficacy and safety of this rapidly evolving field of glaucoma treatment. Further comparative studies would also be helpful to evaluate the relative efficacy of different MIGS devices.

## Figures and Tables

**Table 1:** Physical properties of MIGS devices and approved indications for use.

Instrument	Length (mm)	Luminal diameter (*μ*m)	FDA approval	CE mark
*Schlemm's canal devices*				
Trabectome	NA^∗^	NA^∗^	2004	2004
ELT	NA^∗^	NA^∗^	Pending	1998
iStent	1	120	2012	2004
iStent inject	0.36	230	US IDE	2010
Hydrus	8	185–292	US IDE	2011
*Suprachoroidal devices*				
CyPass	6.35	300 (inner)	2016	2009
		510 (outer)		
iStent Supra	4	160	US IDE	2010
*Subconjunctival devices*				
XEN-45	6	45 (inner)	2016	2011
		150 (outer)		
InnFocus	8.5	70	US IDE	2012

ELT: excimer laser trabeculotomy; NA: not available; US IDE: US investigational device exception. ^∗^No device implantation required.

**Table 2:** Efficacy of MIGS devices.

Author	Year	Operation	Number of eyes	Follow-up (months)	Preop IOP (mmHg)	Postop IOP (mmHg)	IOP reduction (mmHg, %)^∗^	Preop meds	Postop meds	Medication reduction (*n*, %)^∗^
Schlemm's canal devices^∗∗^										
*Trabectome*										
Wecker et al. [39]	2016	Trabectome ± CS	60	12	24.5 ± 3.5	15.7 ± 3.4	8.8 (35.9)	2.1 ± 1.3	1.8 ± 1.2	0.3 (14.3)
Lee et al. [38]	2016	Trabectome	17	6	24.4 ± 4.4	16.9 ± 5.1	7.5 (30.7)	3.9 ± 0.8	2.8 ± 1.6	1.1 (28.2)
Jordan et al. [40]	2013	Trabectome ± CS	261	6	24.2 ± 5.5	18.2 ± 6.1	6 (24.8)	2.1 ± 1.3	1.2 ± 1.1	0.9 (42.9)
Bussel et al. [37]	2015	Trabectome	58	12	23.7 ± 5.5	16.2 ± 3.9	7.5 (31.6)	2.8 ± 1.2	2.0 ± 1.3	0.8 (28.6)
		Trabectome ± CS	15	12	20 ± 5.9	15.6 ± 5.1	4.4 (22.0)	2.5 ± 1.5	1.6 ± 1.4	0.9 (36.0)
Maeda et al. [64]	2013	Trabectome	80	6	26.6 ± 8.1	17.4 ± 3.4	9.2 (34.6)	4.0 ± 1.4	2.3 ± 1.2	1.7 (42.5)
Ting et al. [36]	2012	Trabectome	450	12	25.5 ± 7.9	16.8 ± 3.9	8.7 (34.1)	2.7 ± 1.3	2.2 ± 1.3	0.5 (20.9)
		Trabectome	67	12	29 ± 7.5	16.1 ± 4.0	12.9 (44.5)	3.1 ± 1.2	2.2 ± 1.4	0.8 (28.5)
		Trabectome ± CS	263	12	19.9 ± 5.4	15.6 ± 3.2	4.3 (21.6)	2.4 ± 1.1	1.7 ± 1.3	0.8 (31.3)
		Trabectome ± CS	45	12	21.7 ± 8.4	14.2 ± 3.1	7.5 (34.6)	2.5 ± 1.0	1.6 ± 1.3	1.0 (37.9)
Francis et al. [42]	2008	Trabectome ± CS	304	12	20 ± 6.3	15.5 ± 2.9	4.5 (22.5)	2.7 ± 1.1	1.4 ± 1.3	1.2 (45.7)
Minckler et al. [35]	2005	Trabectome	37	12	28.2 ± 4.4^†^	16.3 ± 2.0	11.9 (42.2)	1.2 ± 0.6	0.4 ± 0.6	0.8 (66.7)

*ELT*										
Babighian et al. [49]	2010	ELT	15	24	25.0 ± 1.9	17.6 ± 2.2	7.4 (29.6)	2.2 ± 0.6	0.7 ± 0.8	1.5 (66.8)
Wilmsmeyer et al. [48]	2006	ELT	75	12	23.3 ± 0.6	18.8 ± 0.8	4.5 (19.3)	1.9 ± 0.1	1.8 ± 0.2	0.1 (5.3)
		ELT + CS	60	12	22.4 ± 0.8	16.4 ± 0.4	6.0 (26.8)	1.1 ± 0.2	1.2 ± 0.2	−0.1 (−9.1)

*iStent*										
Vold et al. [15]	2016	2 iStents	54	36	25.5 ± 2.5	14.6	10.9 (42.7)	0	0.1	−0.1
Katz et al. [7]	2015	1 iStent	38	18	19.8 ± 1.3	15.6 ± 1.5	4.2 (21.2)	1.7 + 0.6	0.18	1.5 (89.5)
		2 iStents	41	18	20.1 ± 1.6	13.8 ± 1.3	6.3 (31.3)	1.8 ± 0.5	0.12	1.6 (93.2)
		3 iStents	40	18	20.4 ± 1.8	12.1 ± 1.2	8.3 (40.7)	1.5 ± 0.7	0.08	1.4 (94.7)
Craven et al. [17]	2012	1 iStent + CS	116	24	18.6 ± 3.4	17.1 ± 2.9	1.5 (8.1)	1.6 ± 0.8	0.3 ± 0.6	1.3 (81.3)
Fea [19]	2010	1 iStent + CS	12	16	17.9 ± 2.6	16.6 ± 3.1	1.3 (7.3)	2 ± 0.9	0	2 (100)
Fernandez-Barrientos et al. [13]	2010	2 iStents + CS	17	12	24.2 ± 1.8^†^	17.6 ± 2.8	6.6 (27.3)	1.1 ± 0.5	0	1.1 (100)
Lindstrom et al. [16]	2016	2 iStents	57	18	19.5 ± 1.5	14.4 ± 2.1	5.1 (26.2)	1	0.02	0.98 (98.0)
Tan and Au [23]	2016	1 iStent + CS	41	36	21.2 ± 4.7	17.1 ± 2.4	4.1 (19.3)	2.1 ± 1.0	1.3 ± 1.2	0.8 (38.1)
Donnenfeld et al. [10]	2015	2 iStents	39	36	20.6 ± 2.0	14.2 ± 2.1	6.4 (31.1)	1	0.05	0.95 (95.0)
Fea et al. [20]	2015	1 iStent + CS	10	48	17.8 ± 2.7	15.9 ± 2.3	1.9 (10.7)	1.9 ± 0.9	0.5 ± 0.8	1.4 (74.7)
Neuhann [22]	2015	1 iStent + CS	62	36	24.1 ± 6.9	14.9 ± 2.3	9.2 (38.2)	1.8 ± 0.9	0.3 ± 0.5	1.5 (83.3)
Ahmed et al. [11]	2014	2 iStents	39	12	22.2 ± 2.0	17.1 ± 2.2	5.1 (23.0)	2	1	1 (50.0)
Arriola-Villalobos et al. [12]	2013	2 iStents + CS	20	12	20.0 ± 3.7	16.8 ± 2.2	3.2 (16.0)	1.3 ± 0.7	0.3 ± 0.6	1 (76.9)
Patel et al. [26]	2013	1 iStent ± CS	44	6	21.5	16.7	4.8 (22.3)	2.3	0.6	1.7 (73.9)
Arriola-Villalobos et al. [25]	2012	1 iStent + CS	19	36	19.4 ± 1.9	16.3 ± 4.2	3.2 (16.3)	1.3 ± 0.5	0.8 ± 0.9	0.48 (36.4)
Buchacra et al. [9]	2011	1 iStent	10	12	26.5 ± 7.9	17.0 ± 2.5	9.5 (35.8)	2.9 ± 0.7	1.1 ± 0.6	1.8 (62.1)
Samuelson et al. [18]	2011	1 iStent + CS	111	12	18.4 ± 3.2	16.9 ± 3.0	1.5 (8.2)	1.5 ± 0.6	0.2 ± 0.6	1.3 (86.7)
Vandewalle et al. [24]	2009	1 iStent ± CS	10	12	19.6	15.8	3.8 (19.4)	2.7	1.7	1 (37.0)
Spiegel et al. [27]	2009	1 iStent ± CS	47	12	21.5 ± 3.7	16.9	4.6 (21.4)	1.6 ± 0.8	0.4	1.2 (75.0)

*iStent inject*										
Fea et al. [28]	2014	2 iStent injects	94	12	21.1 ± 1.7	13.0 ± 2.3	8.1 (38.4)	1	0.1	0.9 (91.5)
Arriola-Villalobos et al. [30]	2016	1 iStent inject + CS	11	60	20.4 ± 4.5	16.2 ± 2.3	4.2 (20.5)	1.2 ± 0.8	1.1 ± 0.8	0.1 (7.6)
Voskanyan et al. [29]	2014	2 iStent injects	99	12	22.1 ± 3.3	15.7 ± 3.7	6.4 (29.0)	2.2 ± 0.4	0.3	1.9 (87.3)
Gonnermann et al. [31]	2016	2 iStent injects + CS	25	12	21.3 ± 4.1	14.0 ± 2.3	7.3 (34.0)	2.0 ± 0.9	1.3 ± 1.2	0.7 (36.0)
Klamann et al. [65]	2015	1 iStent inject^††^	17	6	21.2 ± 2.6	14.2 ± 1.4	7 (33.0)	2.2 ± 0.9	0.9 ± 0.6	1.3 (59.8)
		1 iStent inject^††^	15	6	23.8 ± 3.3	15.3 ± 1.1	8.4 (35.5)	2.3 ± 1.2	1.0 ± 0.3	1.3 (55.4)
		1 iStent inject^††^	3	6	28.3 ± 3.2	N.A.	N.A.	N.A.	N.A.	N.A.

*Hydrus*										
Pfeiffer et al. [32]	2015	Hydrus + CS	50	24	18.9 ± 3.3	16.9 ± 3.3	2.0 (10.6)	2.0 ± 1.0	0.5 ± 1.0	1.5 (75.0)
Fea et al. [33]	2016	Hydrus	31	12	23.1 ± 5.1	16.5 ± 2.6	6.6 (28.5)	2.3 ± 0.8	0.9 ± 1.0	1.4 (60.7)
Gandolfi et al. [34]	2016	Hydrus	21	24	26.0 ± 4.0	16.0 ± 2.0	10.0 (38.5)	2.7	0.9 ± 1.0	1.8 (66.9)

Suprachoroidal devices										
*CyPass*										
Vold et al. [15]	2016	CyPass + CS	374	24	24.4 ± 2.8^†^	17.0 ± 3.4	7.4 (30.3)	1.4 ± 0.9	0.2 ± 0.6	1.2 (85.7)
Hoeh et al. [53]	2016	CyPass + CS	142	12	20.2 ± 6.0	15.9 ± 3.1	4.3 (21.3)	2.0 ± 1.1	1.1	0.9 (45.0)
Garcia-Feijoo et al. [54]	2015	CyPass	65	12	24.5 ± 2.8	16.4 ± 5.5	8.1 (33.1)	2.2 ± 1.1	1.4 ± 1.3	0.8 (36.4)
Hoeh et al. [52]	2013	CyPass + CS	184	6	21.1 ± 5.9	15.6 ± 0.5	5.5 (26.1)	2.1 ± 1.1	0.8	1.35 (64.3)

Subconjunctival devices										
*XEN-45*										
Perez-Torregrosa et al. [55]	2016	XEN-45 + CS	30	12	21.2 ± 3.4	15.0 ± 2.5	6.2 (29.1)	3.1 ± 0.7	0.2 ± 0.7	2.9 (94.5)

*InnFocus*										
Batlle et al. [63]	2016	InnFocus ± CS	23	36	23.8 ± 5.3	10.7 ± 3.5	13.1 (55.0)	2.4 ± 1.0	0.7 ± 1.1	1.7 (70.8)

CS: cataract surgery; ELT: excimer laser trabeculotomy; IOP: intraocular pressure; ∗ represents unpaired results; ∗∗ represents only prospective case series and randomized controlled trials are included; † represents postwashout IOP; †† represents study was split into three groups: phakic open-angle glaucoma, phakic pseudoexfoliation glaucoma, and pseudophakic subjects, respectively.

**Table 3:** Safety of MIGS devices.

Author	Year	Postoperative complications (*n*, %)	Reoperations (*n*, %)
Sclemm's canal devices^∗^			
*Trabectome*			
Lee et al. [38]	2016	Herpetic keratitis reactivation = 1 (5.3)	AC washout = 1 (5.3)
		IOP > 21 mmHg = 5 (26.3)	Repeat glaucoma surgery = 2 (10.5)

Bussel et al. [37]	2014	Transient hypotony = 5 (6.8)	Trabeculectomy = 4 (5.5)
			Tube surgery = 6 (8.2)
			Cyclophotocoagulation = 2 (2.7)
			Repeat Trabectome = 1 (1.4)

Jordan et al. [40]	2013	Reflux bleeding = 512 (91.9)	AC lavage = 2 (0.4)
		IOP > 30 mmHg = 44 (7.8)	
		Cystoid macular edema = 3 (0.5)	

Maeda et al. [64]	2013	Reflux bleeding = 80 (100)	Repeat glaucoma surgery = 13 (16.3)
		Reoperation (glaucoma surgery) = 13 (16.3)	

Ting et al. [36]	2012	Reflux bleeding = 692 (96.5)	Repeat glaucoma surgery = 190 (23.0)
		Early hypotony = 5 (0.6)	

Francis et al. [42]	2008	Transient hypotony = 4 (1.3)	Trabeculectomy = 7 (2.3)
		IOP > 10 mmHg from baseline = 32 (10.5)	Tube surgery = 1 (0.3)
		Reflux bleeding = 238 (78.3)	
		SLT = 1 (0.3)	

Minckler et al. [66]	2005	Reflux bleeding = 37 (100)	0
		Persistent minimal DM injury = 1 (2.7)	
		Peripheral anterior synaechiae = 9 (24.3)	
		Goniosynechiae = 5 (13.5)	
		IOP > 5 mmHg from baseline = 2 (5.4)	

Babighian et al. [49]	2010	IOP > 5 mmHg from baseline = 3 (20)	0

Wilmsmeyer et al. [48]	2006	Iris adhesion to corneal tunnel = 2 (1.5)	Repeat glaucoma surgery = 25 (18.6)
		Fibrin reaction = 3 (2.2)	
		CRVO = 1 (0.7)	

*iStent*			
Tan and Au [23]	2016	Transient hyphema = 1 (2.4)	0

Neuhann [22]	2015	Stent not visible = 1 (1.6)	Cyclophotocoagulation = 3 (4.8)
		Posterior capsular opacification = 1 (1.6)	Shunt surgery = 2 (3.2)

Katz et al. [7]	2015	0	0

Vold et al. [15]	2016	Intraoperative stent malposition = 2 (3.7)	0
		Transient hyphema = 1 (1.9)	
		Intraoperative iridodialysis = 1 (1.9)	

Lindstrom et al. [16]	2016	0	0

Fea et al. [20]	2015	0	

Donnenfeld et al. [10]	2015	Hyphema = 2 (5.1)	AC paracentesis = 1 (2.6)

Ahmed et al. [11]	2014	Transient hypotony = 1 (2.6)	0
		Progression of cataract = 4 (10.3)	
		Corneal ulcer = 1 (2.6)	

Arriola-Villalobos et al. [12]	2013	0	0

Craven et al. [17]	2012	Stent obstruction = 5 (4.3)	Trabeculoplasty = 1 (0.9)
		Stent malposition = 3 (2.6)	Stent repositioning = 3 (2.6)
		YAG laser for stent obstruction = 1 (0.9)	Stent replacement = 1 (0.9)
		Focal argon laser photocoagulation = 1 (0.9)	

Patel et al. [26]	2013	Hyphema = 1 (2.3)	0
Arriola-Villalobos et al. [25]	2012	0	0

Buchacra et al. [9]	2011	Stent malposition = 1 (10.0)	0
		Mild hyphema = 7 (70.0)	
		IOP ≥ 30 mmHg = 1 (10.0)	
		Corneal edema = 2 (20.0)	
		Stent obstruction by blood clot = 3 (30.0)	

Samuelson et al. [18]	2011	Stent obstruction = 4 (4.0)	Stent repositioning = 3 (3.0)
		Stent malposition = 3 (3.0)	Stent replacement = 1 (1.0)
		Elevated IOP = 2 (2.0)	
		Elevated IOP requiring treatment = 1 (1.0)	
		YAG laser for stent obstruction = 1 (1.0)	

Fernandez-Barrientos et al. [13]	2010	Stent malposition = 6 of 34 stents (17.6)	0
		Stent fall out = 1 of 34 stents (2.9)	

Spiegel et al. [27]	2009	Stent malposition (no repositioning) = 6 (10.3)	Trabeculectomy = 2 (3.4)
		Stent obstruction = 7 (12.1)	Stent repositioning = 1 (1.7)
		Argon laser = 1 (1.7)	Stent replacement = 2 (3.4)
			Corneal paracentesis = 1 (1.7)

Vandewalle et al. [24]	2009	Stent malposition = 1 (10.0)	0
		Corneal erosion = 2 (20.0)	
		Blood reflux into angle = 5 (50.5)	

Voskanyan et al. [29]	2014	Elevated IOP requiring medications = 10 (10.1)	Trabeculectomy = 1 (1.0)
		YAG laser for stent obstruction = 2 (2.0)	Phacotrabeculectomy = 1 (1.0)
		Argon laser for stent obstruction = 1 (1.0)	Goniotrephenation = 1 (1.0)
		Stent obstruction = 3 (3.0)	Deep sclerectomy = 1 (1.0)
		Stent malposition = 1 (1.0)	
		Goniosynechiae (without treatment) = 1 (1.0)	
		Lens-iris synechiae (laser treatment) = 1 (1.0)	
		Stent not visible upon gonioscopy = 13 (13.1)	

Arriola-Villalobos et al. [30]	2016	0	0

*Hydrus*			
Fea et al. [33]	2016	Transient IOP spike = 2 (6.5)	0
		BCVA < 2 lines from baseline = 3 (9.7)	

Gandolfi et al. [34]	2016	Transient hyphema = 4 (19.0)	0
		IO ≥ 30 mmHg within 48 hrs = 1 (4.8)	
		YAG lysis of PAS = 4 (19.0)	

Pfeiffer et al. [32]	2015	IOP > 10 mmHg from baseline = 2 (4.0)	Repeat glaucoma surgery = 1 (2.1)
		Focal PAS = 9 (18.8)	

Suprachoroidal devices			
*CyPass*			
Vold et al. [51]	2016	Corneal abrasion = 7 (1.9)	Secondary ocular surgical intervention = 20 (5.5)
		Corneal edema = 13 (3.5)	
		Cyclodialysis cleft > 2 mm circumference = 7 (1.9)	
		Iritis = 32 (8.6)	
		Hypotony = 11 (29.9)	
		IOP ≥ 10 mmHg above baseline = 16 (4.3)	
		Cystoid macular edema = 6 (1.3)	
		Stent obstruction = 8 (2.1)	
		Conjunctivitis = 4 (1.0)	
		Visual field loss progression = 25 (6.7)	

Garcia-Feijoo et al. [54]	2015	IOP > 30 mmHg = 7 (10.8)	Trabeculectomy = 9 (13.8)
		Transient hyphema = 4 (6.2)	Additional CyPass = 2 (3.1)
		BCVA reduced by ≥2 lines = 2 (3.1)	
		Laser trabeculoplasty = 1 (1.6)	

Hoeh et al. [53]	2016	Early postoperative IOP elevation = 2 (1.2)	Repeat glaucoma surgery = 10 (6.0)
		Late postoperative IOP elevation = 3 (1.8)	Implant reposition = 1 (0.6)
		Mild transient hyphema = 2 (1.2)	Implant explantation = 1 (0.6)
		Hypotony = 23 (13.8)	
		Endothelial touch = 2 (1.2)	
		Implant obstruction = 9 (5.4)	
		Macular edema = 1 (0.6)	

Hoeh et al. [52]	2013	AC reaction > 1 month = 8 (4.4)	Device repositioning = 1 (0.6)
		Early hypotony (<1 month) = 25 (13.8)	Repeat glaucoma surgery = 9 (5.0)
		Hypotony > 1 month = 1 (0.5)	
		Shallow AC without central touch = 1 (0.5)	
		IOP > 10 mmHg from baseline = 19 (10.5)	
		Postoperative hyphema = 2 (1.1)	

Subconjunctival devices			
*XEN-45*			
Perez-Torregrosa et al. [55]	2016	Encapsulation of filtration bleb = 1 (3.3)	0

*InnFocus*			
Batlle et al. [63]	2016	Tube-iris touch = 3 (13.0)	Repeat glaucoma surgery = 1 (4.3)
		Transient hypotony < 3 months = 3 (13.0)	AC paracentesis = 1 (4.3)
		Shallow or flat AC = 3 (13.0)	
		Hyphema = 2 (8.7)	
		Choroidal effusion/detachment = 2 (8.7)	
		Elevated IOP requiring bleb needling = 1 (4.3)	
		Tube obstruction = 1 (4.3)	
		Vitreous hemorrhage = 1 (4.3)	
		Bleb leak = 1 (4.3)	

AC: anterior chamber; BCVA: best corrected visual acuity; CRVO: central retinal vein occlusion; IOP: intraocular pressure; PAS: peripheral anterior synechiae; SLT: selective laser trabeculoplasty. ^∗^Only prospective case series and randomized controlled trials are included.
